# The producer cell type of HSV-1 alters the proteomic contents and infectious capacity of virions

**DOI:** 10.1128/jvi.00829-25

**Published:** 2025-08-19

**Authors:** Gary Dunn, Luke F. Domanico, Matthew P. Taylor

**Affiliations:** 1 Microbiology and Cell Biology, Montana State University 33052, Bozeman, Montana, USA; Lerner Research Institute, Cleveland Clinic, Cleveland, Ohio, USA

**Keywords:** interferon, mass spectrometry, producer cell, herpes simplex virus

## Abstract

The cell that a virus replicates in, that is, the producer cell, can alter the macromolecular composition and infectious capacity of the virions that are produced. Herpes simplex virus type 1 (HSV-1) primarily infects keratinocytes of the epidermis or oral mucosa prior to establishing latency in neurons of the peripheral nervous system, where the virus can persist for the lifetime of the host. Many cell lines that are used to amplify HSV-1 are derived from species and tissue types that are less physiologically relevant to HSV-1 disease. To understand whether the producer cell type influences HSV-1 infection, we tested the infectivity of HSV-1 derived from immortalized African green monkey kidney cells (Vero), immortalized human keratinocytes (HaCaT), and primary human foreskin fibroblasts (HFF-1). We observed that the producer cell type alters the capacity of HSV-1 to produce viral transcripts, proteins, and infectious virions from infected cells and susceptibility to inhibition of replication by interferon treatment. HaCaT-derived HSV-1 consistently exhibited enhanced replication over HFF-1 or Vero-derived virus. To determine whether the producer cell type changes the protein composition of virions, we performed an untargeted LC/MS-MS analysis of virions purified from each cell line. Comparison of virion-associated proteins revealed quantitative differences in composition of both cellular and viral proteins, including ICP0, pUL24, and pUL42. These results highlight the influence that the producer cell type has on HSV-1 infection outcomes and suggest that cell-type-specific factors can alter HSV-1 and impact viral replication.

**IMPORTANCE:**

Approximately 67% of the human population harbors herpes simplex virus type 1 (HSV-1) infection. To study HSV-1, laboratories utilize several different cell lines to propagate HSV-1 for downstream experiments. The type of cell used to produce a virus, that is, the producer cell type, can alter the macromolecular composition, immunogenicity, and infectivity of the virions that are produced across several virus families. We found that the producer cell type of HSV-1 alters virion infectivity and virion protein composition. Therefore, the producer cell type may have implications in the spread of HSV-1 and subsequent disease outcomes in humans. Our results also raise concerns about how the use of different cell types to propagate HSV-1 may alter the outcome, interpretation, and reproducibility of experimental results.

## INTRODUCTION

Herpes simplex virus type 1 (HSV-1) inherently relies upon the resources of a host cell to replicate and spread. HSV-1 possesses a broad tropism both *in vitro* and *in vivo*, meaning it can successfully replicate and produce infectious virions from multiple cell types. Different cell types express distinct transcriptomes and proteomes depending on multiple factors that include the function of the cell, the extracellular environment, and cell-cell signaling. Because HSV-1 infects multiple cell types, the virus must contend with these dissimilar host environments to successfully replicate. The state of the cellular environment can change fundamental aspects of viral infection and influence the macromolecular composition and infectious properties of the virions that are produced (1–8). An example of this phenomenon has been described for the gammaherpesvirus Epstein-Barr virus (EBV) (9). The incorporation of viral glycoprotein complexes into the viral envelope of EBV changes based on whether the virus was produced from B cells or epithelial cells, likely due to the expression of MHC II in B cells. The change in glycoprotein incorporation results in altered cellular tropism of EBV. Beyond members of the *Herpesviridae*, producer cell-dependent effects have been described for members of at least five other viral families, eliciting changes in cellular tropism, immune responses, and replicative capacity (1). It is likely that producer cell-dependent effects remain to be described for a wider range of viruses.

There are many cell lines used to propagate HSV-1 for laboratory experiments that study infection and replication. Commonly used cell lines include Vero, BHK-21, and HeLa among others (10). In this paper, we will refer to the type of cell used to propagate HSV-1 as the producer cell type. The producer cell type of HSV-1 has previously been described to alter virion protein composition. Evaluation of HSV-1 virions produced from Vero and HEp-2 cells contained ICP0 and ICP4, but these proteins were not detected in BHK21-derived virions (11). Although ICP0 and ICP4 expression are critical to the establishment of successful HSV-1 infection, it is currently unknown whether their absence in virions alters virion infectivity. Indeed, the effects that the producer cell type has on HSV-1 infectivity remain understudied. To test our hypothesis that the producer cell type of HSV-1 alters virion infectivity, we propagated HSV-1 from immortalized human keratinocytes (HaCaT), African green monkey kidney cells (Vero), and human foreskin fibroblasts (HFF-1). Vero cells were selected because of their prevalent use as a producer cell line for HSV-1 research (12). HaCaT and HFF-1 were chosen because they are closer to a physiologically representative cell type that HSV-1 would naturally encounter (13–15). We then compared the capacity of HSV-1 produced from each cell line to replicate. Our results show that the producer cell type impacts the subsequent detection of viral transcripts, expression of viral proteins, production of infectious virions, and the capacity to replicate in cells stimulated by interferon in all infected cell types that were tested. Both the multiplicity of infection (MOI) and the infected cell type influenced the degree to which the producer cell type altered viral replication. To begin characterizing the molecular features that lead to these producer cell-dependent differences, we performed an untargeted analysis of the proteomic composition of HSV-1 virions. We observed that proteomic composition is dependent on the producer cell type, observing changes in the content of both viral and cellular proteins present in purified virions.

## RESULTS

### Producer cell-dependent effects on genome:PFU of HSV-1

Replication often produces non-infectious virions. Despite their lack of infectivity, non-infectious virions can still influence the replication of infectious virions. Whether non-infectious virions enhance or hinder viral replication is dependent on several factors, including the type of virus and the viral strain (16–19). We hypothesized that the producer cell type of HSV-1 alters the ratio between the number of viral genomes, a proxy number for total virions, and the number of plaque-forming units (PFU) (genome:PFU). To test this hypothesis, we propagated HSV-1 McKrae viral stocks from HaCaT (HaCaT-HSV), Vero (Vero-HSV), and HFF-1 (HFF-HSV) cells. Each viral stock was propagated in triplicate. We used a plaque assay to determine the viral titer of HaCaT-HSV, Vero-HSV, and HFF-HSV and quantitative PCR (qPCR) to determine the genome content of HSV-1 derived from each producer cell type. The genome:PFU of each producer cell type was quantified specifically for the viral stocks that were used to infect target cells for the experiments described in this paper. HaCaT-HSV had the lowest genome:PFU ratio in comparison to Vero-HSV and HFF-HSV (Table 1). The data support the conclusion that the producer cell type alters the genome:PFU of viral stocks. The subsequent experiments discussed in this paper rely upon infection of target cells using an equivalent MOI based on the PFU applied per cell. It is important to note that normalizing MOI to the genome content of the viral stocks rather than the PFU/cell would result in a lower PFU/cell applied to target cells that were infected with Vero-HSV or HFF-HSV relative to cells infected with HaCaT-HSV. Previous work has shown that higher MOI infections result in enhanced replication of HSV-1 (20, 21).

**TABLE 1:** Producer cell-dependent effects on the HSV-1 genome to PFU ratio^*a*^

HSV-1 producer cell	Average genome:PFU ratio	HSV-1 infectious MOI when normalized to HaCaT-HSV genome content
HaCaT-HSV	4.6 ± 0.8	10.0
Vero-HSV	14.4 ± 1.8^**^	3.0
HFF-HSV	17.0 ± 3.4^**^	3.0

^*a*^ The genome to PFU ratio of HSV-1 derived from HaCaT, Vero, or HFF-1 biological triplicate stocks used for experiments. Virion genome content was determined using qPCR on stocks subjected to prior DNAase digestion to eliminate non-virion genomes. PFUs were quantified by plaque assay on Vero cells. All values are presented with ± the SEM within triplicate samples. Analysis of the statistical significance was performed relative to HaCaT-HSV using an unpaired t-test. ** indicates *P* < 0.01.

### Producer cell-dependent effects on HSV-1 transcription

**Fig 1:**
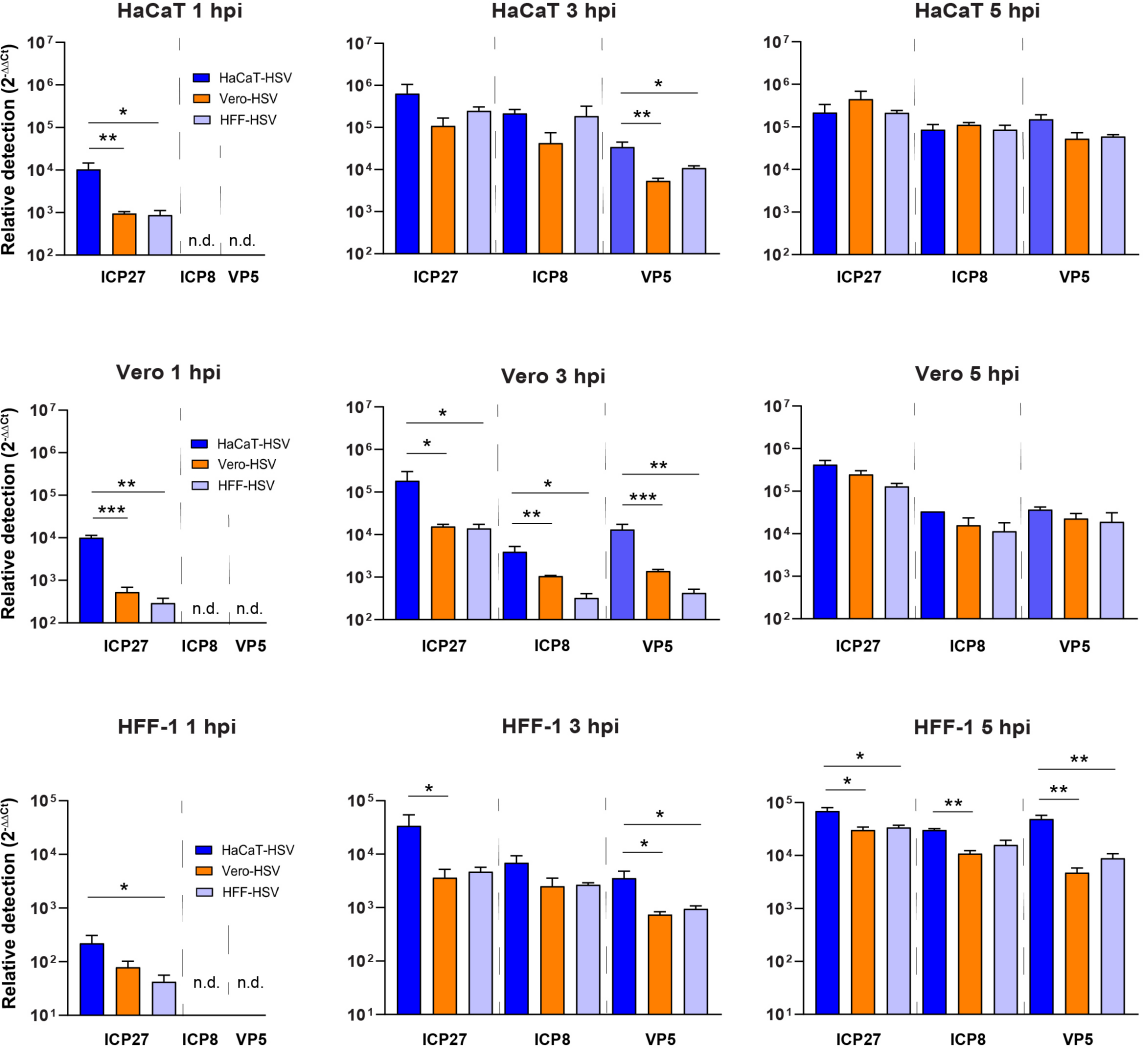
Producer cell-dependent effects on HSV-1 gene expression. HaCaT, Vero, and HFF-1 cells were infected with HSV-1 McKrae derived from HaCaT, Vero, or HFF-1 cells at an MOI of 10. Infected cells were harvested at 1, 3, and 5 hpi for RT-qPCR of ICP27, ICP8, and VP5 transcripts. Relative quantification of RNA was performed using the ΔΔCt method. 28S rRNA was used as an endogenous control. qRT-PCR data are representative of three biological replicates with error bars representing SEM. n.d. means no detection. * indicates *P* < 0.05, ** indicates *P* < 0.01, *** indicates *P* < 0.001 using one-way ANOVA with Tukey’s multiple comparisons test.

To determine whether HSV-1 gene transcript abundance changes based on the HSV-1 producer cell type, HaCaT, Vero, and HFF-1 cells were infected with HSV-1 McKrae at an MOI of 10 derived from HaCaT, Vero, or HFF-1 cells. qRT-PCR was used to quantify viral transcripts of immediate-early (IE) ICP27, early (E) ICP8, and late (L) VP5 relative to cellular 28S rRNA. Viral RNA abundance was measured at 1, 3, and 5 hours post-infection (hpi). HaCaT-HSV produced significantly more ICP27 transcripts at 1 hpi (all cell types), 3 hpi (Vero and HFF-1), and 5 hpi (HFF-1) relative to Vero-HSV and HFF-HSV (Fig. 1). The E-transcript ICP8 only showed significant differences for HaCaT-HSV at 3 hpi in Vero cells. By contrast, the L-transcript VP5 was more abundant following HaCaT-HSV infection in all cell types at 3 hpi. No significant difference in any HSV-1 transcript was observed in either Vero or HaCaT cells by 5 hpi. However, ICP27, ICP8, and VP5 were each detected in greater quantities at 5 hpi in HFF-1 cells infected by HaCaT-HSV relative to Vero-HSV and HFF-HSV. These results show that the abundance of viral transcripts synthesized by HSV-1 McKrae is producer cell-dependent, and that the effect on transcription can depend on the infected cell type and the specific gene that is tested.

### Producer cell-dependent effects on HSV-1 protein expression

**Fig 2:**
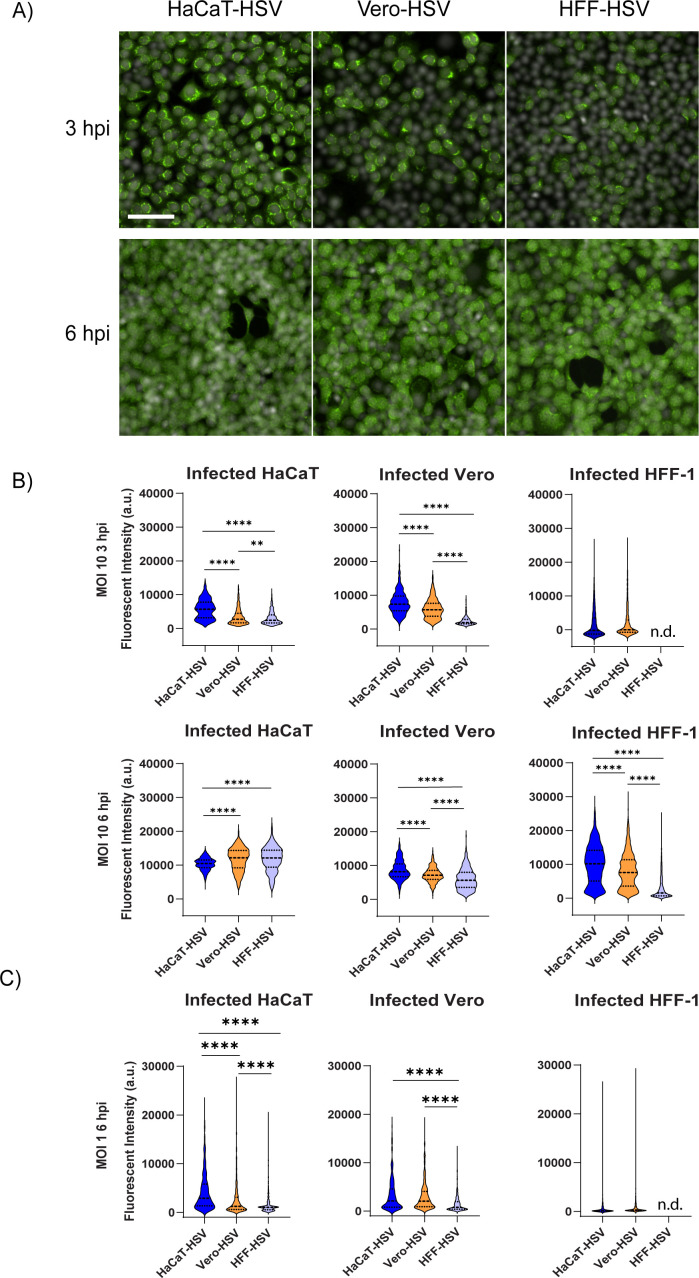
HaCaT-HSV produces more viral proteins early in infection. (**A**) Images of HaCaT cells infected with HSV-1 McKrae derived from HaCaT, Vero, or HFF-1 cells at an MOI of 10. Infected HaCaTs were fixed at 3 and 6 hpi and stained with rabbit polyclonal HSV-1 anti-serum (green) and Hoechst (white). Equivalent exposure and pixel intensity scales are applied for each channel across all presented images. Scale bar = 200 µm. (**B**) Quantification of fluorescent intensity of viral proteins in HaCaT, Vero, or HFF-1 cells infected with HSV-1 McKrae derived from HaCaT, Vero, or HFF-1 at an MOI of 10 or (**C**) MOI 1. Infected cells were fixed and stained as described in A at 3 hpi (B, top row) and 6 hpi (B, bottom row, and C). Quantification of fluorescent intensity was performed using Image J. n.d. = no detection. Statistical comparison was performed using one-way ANOVA with Tukey’s multiple comparisons test. ** indicates *P* < 0.01, **** indicates *P* < 0.0001.

To determine whether the producer cell type alters the capacity of virions to express viral proteins, HaCaT, Vero, and HFF-1 cells were infected at an MOI of 1 and 10 with HSV-1 propagated from the aforementioned cell types. Both MOIs were tested to evaluate the effect of infectious dose on subsequent producer-dependent effects. Infected cells were fixed at 3 hpi for MOI 10 or 6 hpi for MOI 1 and 10. Detection of viral proteins was achieved by staining with a polyclonal HSV-1 antiserum (Fig. 2A). Except for 6 hpi in HaCaTs, HaCaT-HSV consistently produced more detectable viral proteins in cells when infected at an MOI of 10 (Fig. 2B). At an MOI of 1, HaCaT-HSV produced a greater quantity of viral proteins in HaCaTs but produced equivalent amounts of protein compared to Vero-HSV in Vero and HFF-1 cells (Fig. 2C). HFF-HSV consistently produced the lowest quantity of detectable viral proteins in all treatment conditions and did not produce detectable amounts of protein in HFF-1 cells at an MOI of 1 at either time point. In the cell types that were tested, these results show that HSV-1 produced from HaCaT cells expresses greater amounts of detectable viral proteins compared to HSV-1 derived from Vero or HFF-1 cells.

### Producer cell-dependent effects on the production of infectious HSV-1

To determine whether the producer cell type of HSV-1 alters the quantity of infectious virions produced in subsequently infected cells, HSV-1 propagated from the aforementioned cell types was inoculated onto HaCaT, Vero, and HFF-1 cells at an MOI of 1 or 10. The amount of infectious virus that was produced at the indicated time points from infected cells was determined by plaque assay. Each cell line exhibited differences in total virion productivity when averaging the total PFU produced per cell from all infections by HaCaT-HSV, Vero-HSV, and HFF-HSV. When infected at an MOI of 10, we observed on average 8, 22, and 43 PFU/cell produced from Vero, HaCaT, and HFF-1 cells, respectively, at 24 hpi. When cells were infected at an MOI of 1, we observed on average 72 and 46 PFU/cell produced from HaCaT and HFF-1 cells, respectively. Therefore, independent of the source of inoculating material, we observed differences in the per-cell productivity of infectious virus. These differences are an additional reflection on virion productivity and subsequent impact on infectious material.

**Fig 3:**
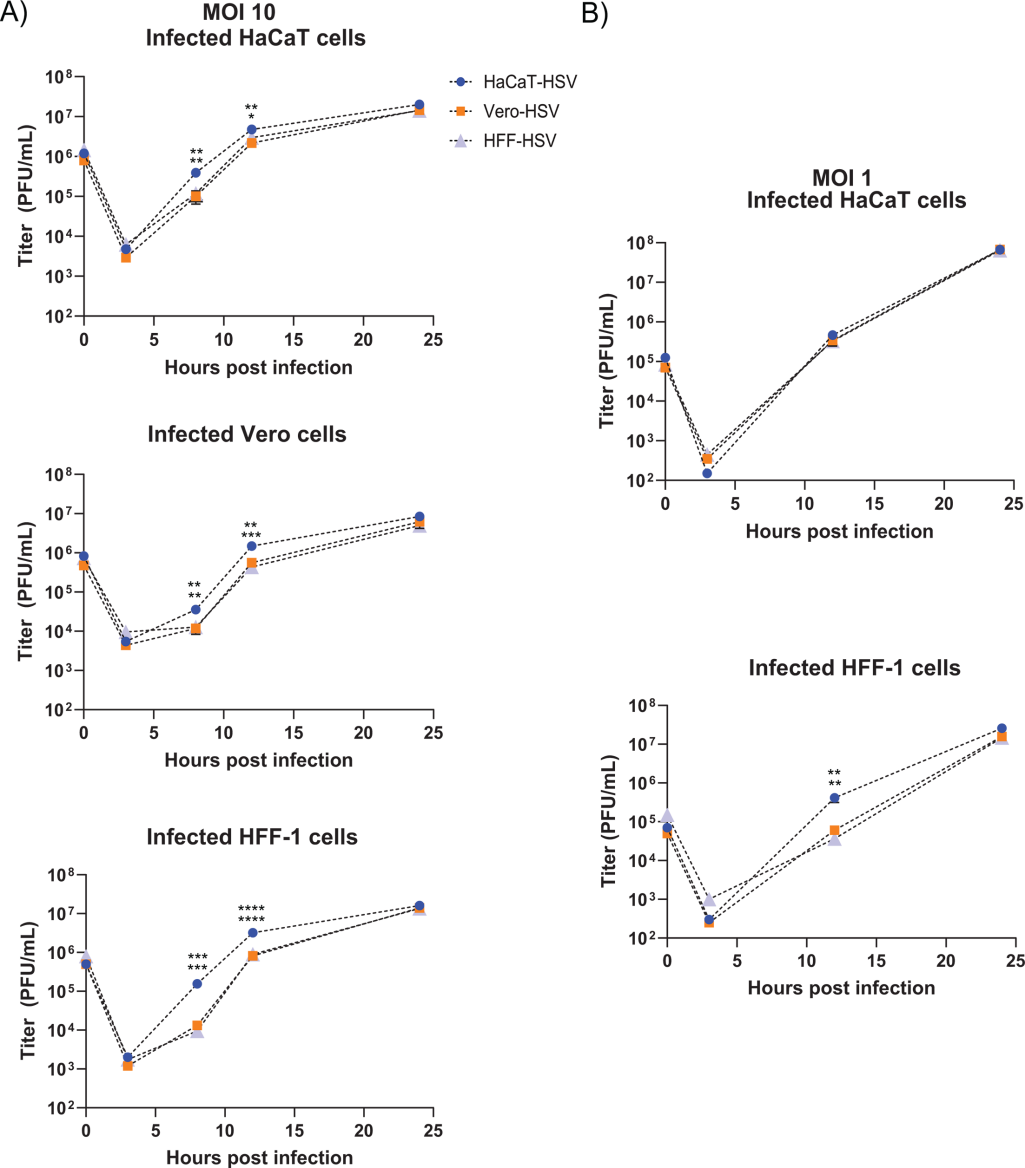
HaCaT-HSV produces higher viral titers early in infection. HaCaT, Vero, and HFF-1 cells were infected with HSV-1 McKrae derived from HaCaT, Vero, or HFF-1 cells at an MOI of 10 (**A**) or 1 (**B**). Virally infected cells and supernatant were harvested at corresponding time points after infection and titered using plaque assay on Vero cells. The values represent means from three biological replicates per treatment across two experiments (MOI 10) or one experiment (MOI 1). Average cell numbers per well for each experiment were 33,750 (HFF-1), 86,250 (HaCaT), and 87,250 (Vero). Error bars indicate the SEM. * indicates *P* < 0.05, ** indicates *P* < 0.01, *** indicates *P* < 0.001, **** indicates *P* < 0.0001 using one-way ANOVA with Tukey’s multiple comparisons test. Statistical comparison shows HaCaT-HSV relative to Vero-HSV (upper asterisks) and HaCaT-HSV relative to HFF-HSV (lower asterisks).

HaCaT-HSV inoculation consistently produced higher quantities of infectious HSV-1 in HaCaT, Vero, and HFF-1 cells at 8 and 12 hpi in comparison to Vero-HSV and HFF-HSV at an MOI of 10 (Fig. 3A). When HFF-1 cells were infected at an MOI of 1, HaCaT-HSV produced more virions at 12 hpi only, and production of virions was equivalent in all time points tested in HaCaT cells at MOI 1 (Fig. 3B). The output of infectious virions was roughly equivalent in all tested conditions by 24 hpi. The data show that HaCaT-HSV has a propensity for producing higher quantities of infectious virions in cells infected at MOI 10, but this capacity for enhanced replication partially or completely diminishes when cells are infected with an MOI of 1.

### Producer cell-dependent effects on the sensitivity of HSV-1 to type I and type II interferon

Cells possess a multitude of pathways that detect viral antigens and products of viral replication, leading to the secretion of interferons (IFN) from both infected and uninfected cells (22–31). Over the course of infection, HSV-1 inevitably encounters cells that have entered an antiviral state through the uptake of IFNs, including IFN-γ and IFN-β, which limits viral replication and spread within hosts (32–35). HSV-1 replication is severely dampened in cells pre-treated with type I and type II IFN *in vitro* (33, 36–48). To test whether the producer cell type influences the sensitivity of HSV-1 to IFN responses, we evaluated HSV-1 replication following pre-treatment of cells with IFN-β or IFN-γ. The concentration of IFN required to inhibit viral replication varies between studies, likely in a cell type-dependent manner (36–39, 44). The concentrations of IFN used for these experiments were based on the amount required to maximally inhibit viral replication in each cell type. Target cells were pre-treated for 16 hours with 100 U/mL (HaCaT) or 1,000 U/mL (HFF-1) of IFN-β or IFN-γ prior to infection at an MOI of 10 or 1 with HSV-1 McKrae propagated from the previously identified cell types. HSV-1 was harvested from infected cells for titer at 12 hpi (MOI 10) or 24 hpi (MOI 1). The fold reduction in titer due to IFN treatment was calculated in comparison to untreated control infections for each inoculum in the target cells.

**Fig 4:**
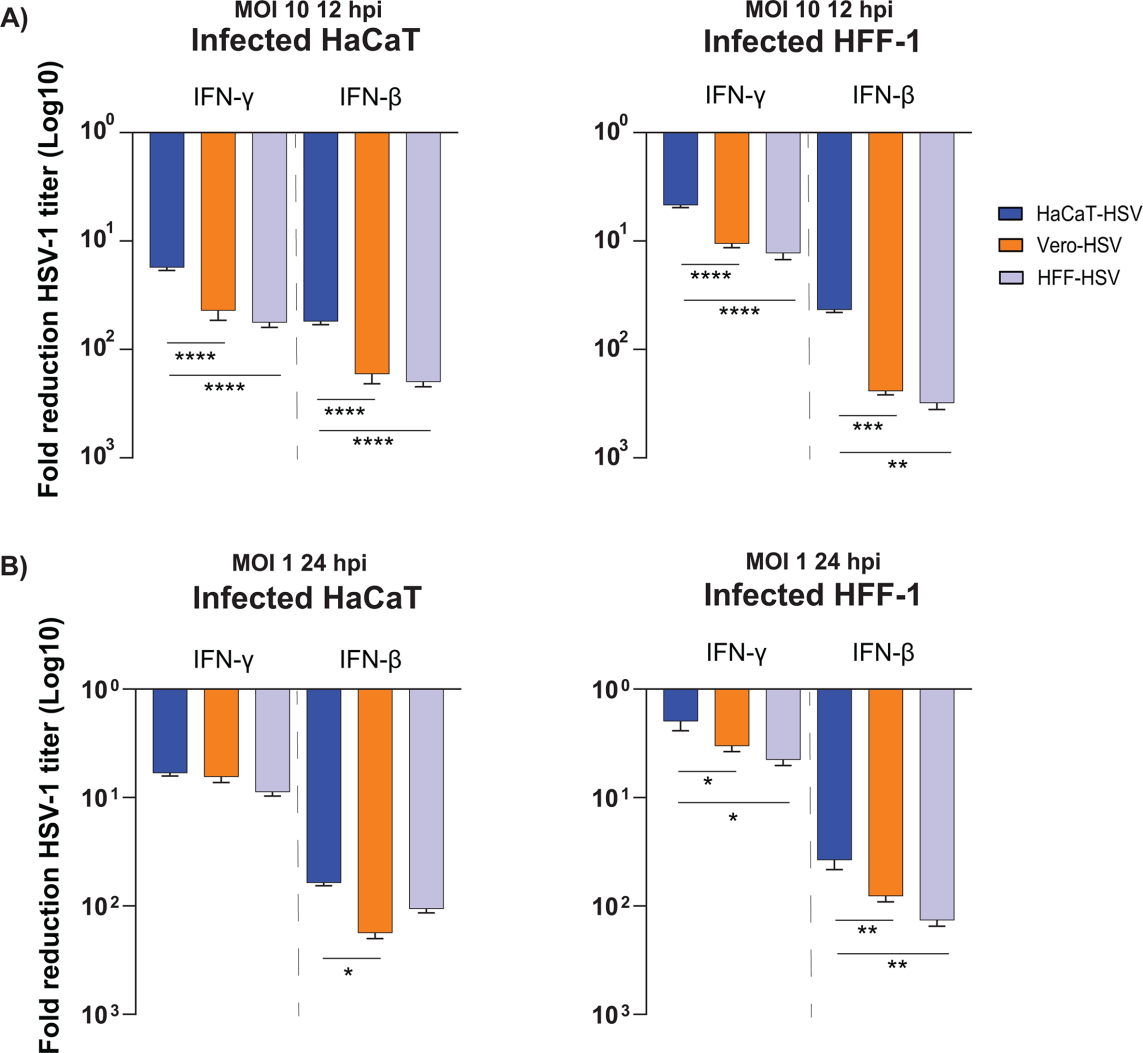
HaCaT-HSV replication is less sensitive to pre-treatment with type I and type II interferon. Cells were pre-treated with 100 U/mL (HaCaT) or 1,000 U/mL (HFF-1) for 16 hours prior to infection with HSV-1 McKrae derived from HaCaT, Vero, or HFF-1 cells at an MOI of 10 or 1. Virally infected cells and supernatant were harvested 12 hpi (MOI 10) or 24 hpi (MOI 1) and titered on Vero cells. The values represent the mean reduction in viral titers across two experiments (MOI 10) or one experiment (MOI 1) from three biological replicates relative to infected cells that did not receive IFN pre-treatment. Average cell numbers per well for each experiment were 36,875 (HFF-1) and 86,150 (HaCaT). Error bars indicate the SEM. Statistical comparison of the producer cell-dependent PFU/cell output between conditions used one-way ANOVA with Tukey’s multiple comparisons test. * indicates *P* < 0.05, ** indicates *P* < 0.01, *** indicates *P* < 0.001, **** indicates *P* < 0.0001.

In both target cell types, IFN-β had a greater inhibitory effect on viral replication based on output viral titer in comparison to IFN-γ. Interestingly, the reduction of HaCaT-HSV replication by IFN pre-treatment was less than the reduction in replication observed for Vero-HSV and HFF-HSV in MOI 10 infected HaCaT and HFF-1 cells (Fig. 4A). Vero-HSV and HFF-HSV exhibited similar reductions to replication in IFN-treated cells relative to one another. Reduced sensitivity of HaCaT-HSV to both IFN-γ and IFN-β inhibition was maintained in HFF-1 cells at an MOI of 1, but MOI 1 infection of HaCaTs resulted in similar reductions in HSV-1 replication regardless of the producer cell type (Fig. 4B). These results show that HSV-1 produced from HaCaT cells is less sensitive to the antiviral effects of type I and II IFN at MOI 10, and that differences in sensitivity at MOI 1 are dependent upon the infected cell type.

### Producer cell-dependent effects on the HSV-1 virion proteome

Our results have shown that HSV-1 replication changes based on the producer cell type of HSV-1 virions. HSV-1 expresses 84 viral proteins during infection that perform a wide array of functions critical to viral infectivity and replication (49). At least 44 of these proteins have been previously identified as being incorporated into mature HSV-1 virions (50–52). We hypothesized that the phenotypes we have observed are due to producer cell-dependent changes to the proteome associated with HSV-1 virions. To test this hypothesis, HSV-1 McKrae was propagated from HaCaT, Vero, and HFF-1 cells, and extracellular virus was subjected to a modified version of a previously described purification protocol (52). Virions purified from each cell line were produced under conditions of serum starvation over the course of viral replication, as we previously observed that serum components can potentially co-purify with HSV-1 virions when visualizing proteins on SDS-PAGE. We did not observe any significant reductions in the titer of virus produced when serum was removed from the media. For purification, secreted HSV-1 virions were separated away from cellular components using a combination of centrifugation, filtration, and DNAse digestion prior to further purification of virions by ultracentrifugation on a 10% Ficoll 400 cushion. Purified virions were then processed for liquid chromatography and tandem mass spectrometry (LC-MS/MS) of tryptic peptides using a Data Independent Analysis (DIA) workflow. Proteins associated with purified HSV-1 virions were identified and quantified to determine which viral and host cellular proteins are differentially detected between the samples. The primary quantification of each protein is presented prior to normalization (Table S1). To determine whether proteins were either virion-associated or contaminating proteins that co-purify with virions, mock-infected cells were scraped and frozen at −80°C. After thawing, the mock-infected cell supernatants were subjected to the same purification scheme, and proteins that were detected from mock-infected cells were identified as potential contaminants and removed from further analysis (Table S3A and B). As an additional control, separate replicates of HaCaT-HSV virions were concentrated and digested with Proteinase K prior to Ficoll separation (53). Proteins that are not contained within lipid bilayers are accessible to Proteinase K digestion, resulting in a reduction in the detection of the associated peptides. We considered many of these proteins, other than those known to be virion envelope proteins, to be either a likely contaminant or partial contaminant, meaning that fractions of the protein were both virion and non-virion associated (Table S2D).

**Fig 5:**
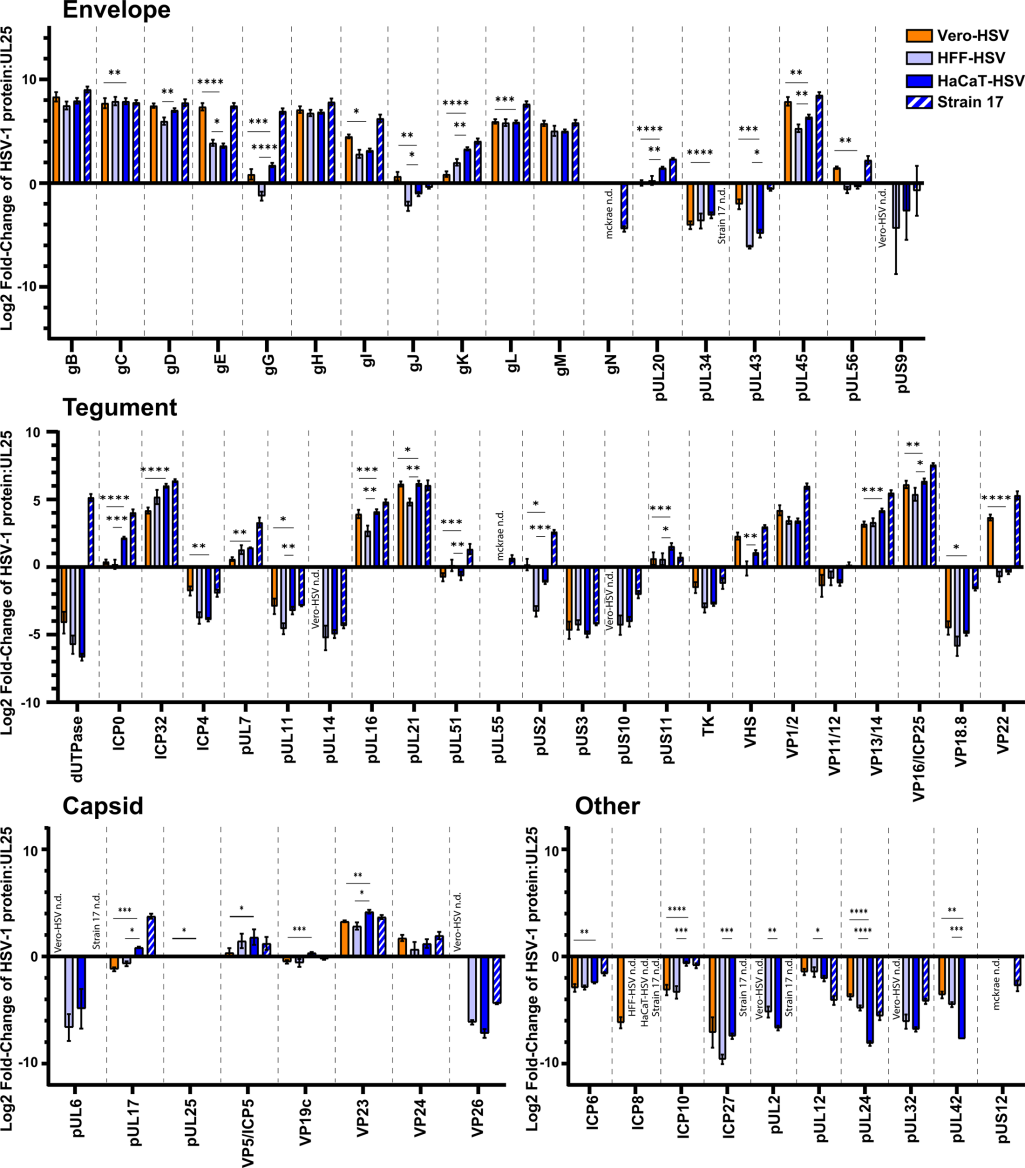
HSV-1 virions incorporate viral proteins in a producer cell and strain-dependent manner. HSV-1 virions were purified from Vero and HFF-1 cells (McKrae) or HaCaT cells (McKrae and strain 17). LC-MS/MS was performed on purified virions using an Orbitrap 480 mass spectrometer, and protein identification and quantification were based on data-independent acquisition. Relative protein abundance of each viral protein is expressed as the average VSN (variance stabilization normalization) Log2 fold-change (FC) relative to pUL25 detected in each sample type. Protein data are organized based on the protein localization within the virion. Error bars indicate the SEM within triplicate samples. n.d. means no detection. Statistical comparison of values employed Empirical Bayes statistics for differential detection test. * Indicates *P* < 0.05, ** indicates *P* < 0.01, *** indicates *P* < 0.001, **** indicates *P* < 0.0001.

**Fig 6:**
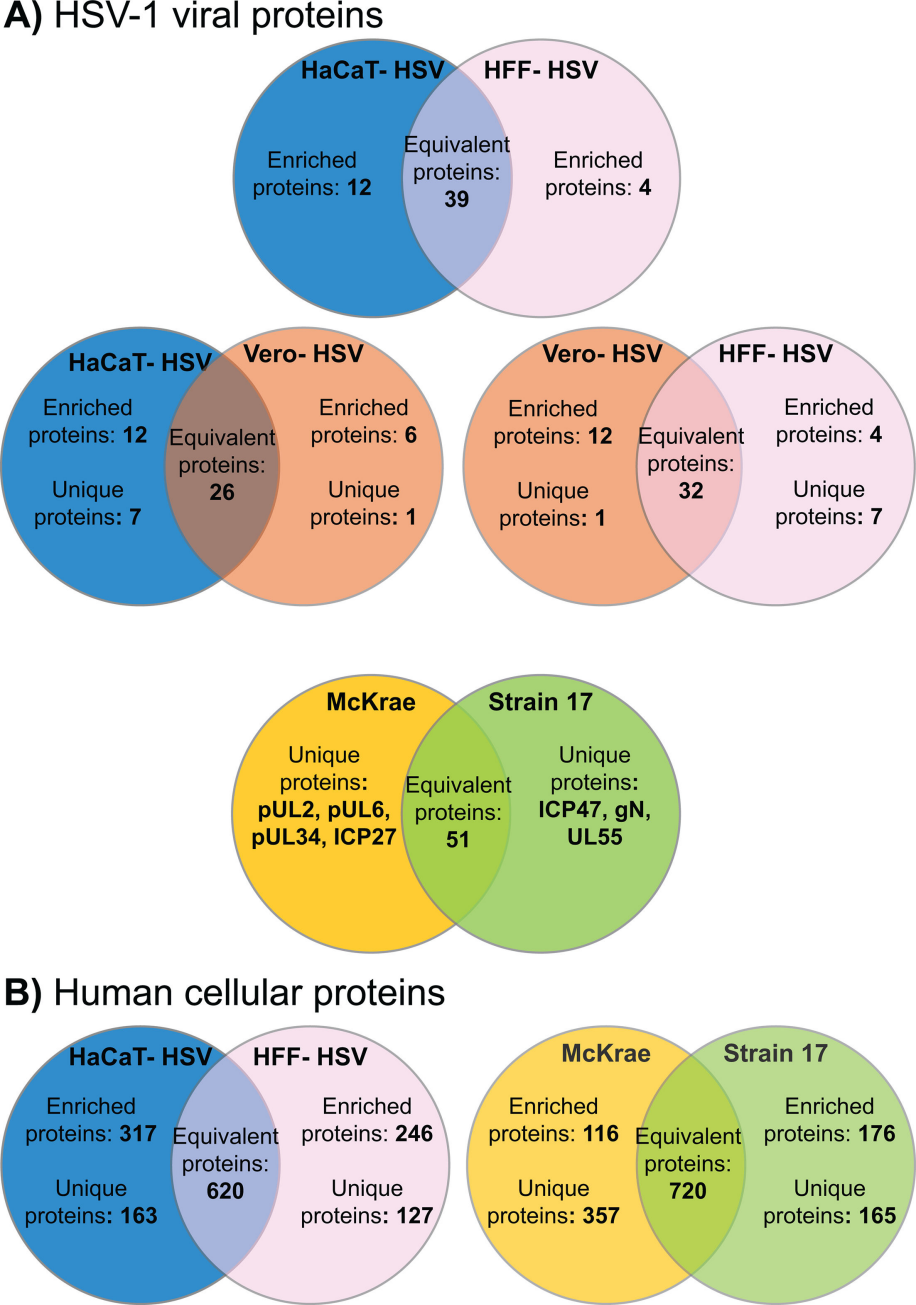
Summary of HSV-1 protein incorporation into virions. Venn diagrams depict commonalities and differences in HSV-1 viral protein (**A**) and cellular protein (**B**) incorporation based on the producer cell type and strain. Strain and cell type being compared are indicated at the top of each diagram. Venn diagrams are not drawn to proportional scale but instead highlight overall viral and cellular protein comparisons between samples. The proteomics data that is summarized in each Venn diagram that compares viral and cellular protein detection in purified virions can be found in Tables S2 and S3. The proteomics data directly compare the detection of viral proteins between HaCaT-HSV and HFF-HSV (Table S2A), HaCaT-HSV and Vero-HSV (Table S2B), and between HSV-1 McKrae and HSV-1 strain 17 (Table S2E). The proteomics data also compare the detection of cellular proteins, after the removal of proteins detected in purified mock and proteinase k-treated samples, within HaCaT-HSV and HFF-HSV (Table S3D through H) as well as HSV-1 McKrae and strain 17 (Table S2E).

LC-MS/MS identified several viral and cellular proteins that were differentially incorporated into HSV-1 virions based on the producer cell type. In total, 56 viral proteins were detected in HSV-1 McKrae. Three proteins, which are not incorporated into the viral envelope, thymidine kinase, pUL7, and pUL51, exhibited reduced detection following Proteinase K digestion (Table S2D) (52). A fourth protein, pUL50 deoxyuridine triphosphatase, was also reduced by Proteinase K digestion, but has not been previously characterized as a tegument protein. Reduced detection suggests that a subset of these proteins is external to the virion envelope. All other Proteinase K accessible viral proteins were envelope proteins, with patterns of reduced detection in line with prior reports of viral membrane proteins (53). A quantitative comparison of viral proteins showed that 25 and 34 proteins were differentially incorporated into HFF-HSV and Vero-HSV, respectively, relative to HaCaT-HSV (Fig. 5; Table S2A through C). When applying a stricter threshold for defining differential protein incorporation (greater than 1 log_2_ fold-change with a *P* < 0.01), 39 out of 55 proteins were found to be equivalently detected between HaCaT-HSV and HFF-HSV compared to 26 out of 56 proteins when comparing HaCaT-HSV to Vero-HSV (Fig. 6A). A total of seven viral proteins were detected in HaCaT-HSV and HFF-HSV that were not detected in Vero-HSV. Some of this lack of detection is likely due to the limits of mass spectrometry, including the limited detection of low molecular weight proteins (54). For example, neither VP26 nor pUs9 was detected in Vero-HSV, and each of these proteins exhibited relatively low or variable detectability between replicate samples in both the HaCaT-HSV and HFF-HSV samples. ICP8 was uniquely detected in Vero-HSV. To address the hypothesis that the virion proteome is contributing to the enhanced infectivity of HaCaT-HSV, we compiled a list of viral proteins that were incorporated into HaCaT-HSV at greater than 1 log_2_ fold-change with a *P* < 0.01 in comparison to both Vero-HSV and HFF-HSV. These proteins included gG, gK, ICP0, and ICP10. We were also interested in viral proteins that may inhibit viral replication. Viral proteins that were incorporated into both Vero-HSV and HFF-HSV at greater than 1 log_2_ fold-change with a *P* < 0.01 in comparison to HaCaT-HSV include pUL24 and pUL42.

A quantitative analysis of human cellular protein incorporation into virions was performed comparing incorporation into HaCaT-HSV relative to HFF-HSV (Table S2B). Comparison of cellular protein incorporation between HaCaT-HSV and Vero-HSV was determined to be unreliable due to protein heterogeneity between species (Table S2C). Across all human-derived samples and controls, 3,225 proteins were identified. A total of 1,061 human proteins were detected in both mock-infected and HSV-1-infected samples. In total, 185 of these 1,061 proteins were detected in greater quantities in viral samples compared to mock. Although some of the cellular proteins identified in mock samples were enriched in viral samples, we decided to treat these as contaminating proteins in the context of our data analysis. Removal of control-detected proteins and low-quality detections left 1,880 cellular proteins uniquely detected in HSV-1-infected samples (Table S3A through C). In addition, 344 human cellular proteins were accessible to proteinase k digestion in HaCaT-HSV, indicative of either their membrane topology or presence as an extra-virion contaminant (Table S2D). There were 620 cell proteins that were equivalently detected between HaCaT-HSV and HFF-HSV (Table S3H). HaCaT-derived HSV-1 contained 163 unique cellular proteins, in comparison to 127 proteins unique to HFF-HSV (Table S3D and F). An additional 365 proteins were enriched in HaCaT-HSV relative to HFF-HSV, compared to 283 proteins enriched in HFF-HSV relative to HaCaT-HSV (Table S3E and G). It is difficult to determine what the role of these proteins is in the context of their association with the virion. However, many of the virion-associated cellular proteins are affiliated with vesicular transport, exocytosis, and metabolic processes, among others. A general summary of the differential detection of viral and cellular proteins based on the producer cell type of HSV-1 is found in Fig. 6A and B.

### Strain-dependent effects on the HSV-1 virion proteome

Several previous studies have used LC-MS/MS to identify HSV-1 virion incorporated proteins. These studies have used different viral strains and producer cell types for virion production and analysis (Table 2) (50–52). Because the incorporation of viral and cellular proteins into HSV-1 virions often varies between studies, we isolated HSV-1 strain 17 from HaCaT cells to compare the proteome of HaCaT-derived McKrae and strain 17 virions.

**TABLE 2:** Detection of HSV-1 proteins in purified HSV-1 across studies^*a*^

HSV-1 gene	HSV-1 protein	HSV-1 protein detection in this study		HSV-1 protein detection from previous studies		
		Strain McKrae (HaCaT, Vero, HFF-1)	Strain 17 (HaCaT)	Strain F (HeLa) (52)	Strain Sc16 (HaCaT) (51)	Strain 17+ (BHK-21) (50)
Envelope						
UL1	gL	+a	+	+	+	+
UL10	gM	+	+	+	+	+
UL20		+a	+	WB	+	+
UL22	gH	+	+	+	+	+
UL27	gB	+	+	+	+	+
UL34		+a	−b	−	+	+
UL43		+a	+	−	−	x
UL44	gC	+a	+	+	+	+
UL45		+a	+	+	+	+
UL49.5	gN	−	+b	−	−	+
UL53	gK	+a	+	−	+	+
UL56		+a,c	+	+	+	+
US4	gG	+a,c	+	+	+	+
US5	gJ	+a	+	−	−	−
US6	gD	+a,c	+	+	+	+
US7	gI	+a,c	+	+	+	+
US8	gE	+a,c	+	+	+	+
US9		+a,c	+	+	−	+
Capsid						
UL6		+a	−b	+	+	+
UL17		+a	+	+	+	+
UL18	VP23	+a	+	+	+	+
UL19	VP5/ICP5	+a	+	+	+	+
UL25		+a	+	+	+	+
UL26	VP24	+	+	+	+	+
UL35	VP26	+a	+	+	+	+
UL38	VP19c	+a	+	+	+	+
Tegument						
Rs1	ICP4	+a	+	+	+	+
RL1	ICP34.5	−	−	+	−	−
RL2	ICP0	+a	+	+	+	+
UL7		+a	+	+	+	+
UL11		+a	+	+	+	+
UL13	VP18.8	+a	+	+	+	+
UL14		+a	+	+	+	+
UL16		+a	+	+	+	+
UL21		+a	+	+	+	+
UL23	TK	+c	+	+	+	+
UL31		−	−	−	+	+
UL36	VP1/2	+	+	+	+	+
UL37	ICP32	+a	+	+	+	+
UL41	VHS	+a	+	+	+	+
UL46	VP11/12	+	+	+	+	+
UL47	VP13/14	+a	+	+	+	+
UL48	VP16/ICP25	+a,c	+	+	+	+
UL49	VP22	+a,c	+	+	+	+
UL50	dUTPase	+	+	+	+	+
UL51		+a	+	+	+	+
UL55		−	+b	+	+	+
US1	ICP22	−	−	−	+	+
US2		+a	+	+	+	+
US3		+	+	+	+	+
US10		+a	+	+	+	+
US11		+a	+	−	+	+
Unknown						
UL2		+a	−b	−	+	+
UL3		−	−	−	−	+
UL4		−	−	−	−	−
UL5		−	−	−	−	+
UL8		−	−	−	−	−
UL9		−	−	−	−	−
UL12		+	+	−	−	+
UL15		−	−	−	−	−
UL24		+a	+	−	−	+
UL28	ICP18.5	−	−	−	−	−
UL29	ICP8	+a	−b	−	−	+
UL30		−	−	−	−	+
UL32		+a	+	−	−	+
UL33		−	−	−	−	−
UL39	ICP6	+a	+	−	+	+
UL40	ICP10	+a	+	−	+	+
UL42		+a	−	−	−	+
UL52		−	−	−	−	+
UL54	ICP27	+a	−b	−	+	+
US12	ICP47	−	+b	−	−	+

^*a*^ Comparison of HSV-1 protein detection by mass spectrometry between our study and previous studies. +, detected in purified HSV-1 virions; −, not detected in purified HSV-1 virions; a, producer cell-dependent incorporation in HSV-1 McKrae (current study); b, strain-dependent effect on viral protein incorporated (current study); c, the viral protein was accessible to proteinase k digestion (current study); WB, the viral protein was detected by Western blot but not by mass spectrometry; x, the viral gene was deleted in the HSV-1 strain used in the study.

A quantitative comparison of HSV-1 McKrae and strain 17 viral protein incorporation was complicated by protein sequence heterogeneity between the two strains. However, LC-MS/MS analysis did reveal a subset of viral proteins that are uniquely incorporated into virions in a strain-dependent manner (Table S2E). In total, 55 and 54 viral proteins were detected in HSV-1 McKrae and strain 17 derived from HaCaT cells, respectively (Fig. 5). ICP47, gN, and pUL55 were each detected in strain 17 but were undetected in McKrae. Conversely, pUL2, pUL6, pUL34, and ICP27 were all detected in McKrae but not strain 17. Human cellular proteins were abundant in both strains. Strain 17 virions contained 1,166 total cellular proteins whereas McKrae contained 1,361 cellular proteins (Table S2E). In all, 720 cellular proteins were equivalently detected between each strain, with 181 and 119 of those proteins enriched (greater than 1 log_2_ fold-change with a *P* < 0.05) in strain 17 and McKrae, respectively. Even though each strain was derived from HaCaT cells, 165 unique proteins were detected in strain 17 compared to 357 unique proteins detected in McKrae. The results from our LC-MS/MS analysis show that both the producer cell type and strain of HSV-1 influence the incorporation of both viral and cellular proteins into virions. A summary of the differential detection of viral and cellular proteins based on the strain is found in Fig. 6A and B.

## DISCUSSION

The producer cell type can alter the infectivity, antigenicity, and cellular tropism of several viruses. Some of these phenotypes have been attributed to producer cell type-dependent changes to the macromolecular contents of the virions themselves. These changes in virion content include altered protein glycosylation patterns, differential protein incorporation, distinct lipid compositions, and selection for genomic mutations. The goal of the work presented here was to determine whether cell type alters the capacity of the produced HSV-1 virions to initiate infection and replicate. The cell types that were selected for these experiments were relevant to HSV-1, including HaCaT and HFF-1 cells. In addition, Vero cells were chosen due to their common use as a producer and experimental cell line for HSV-1. The replication capacity of virions from each producer cell type was assessed on multiple cell lines, as measured by viral transcription, protein production, and the output of infectious virions. When comparing HaCaT-HSV, Vero-HSV, and HFF-HSV infectivity of cells, HSV-1 produced in HaCaT cells exhibited the most robust replication in target cells overall, including cells that had been pre-treated with type I and type II interferon. We hypothesized that the observed differences in HSV-1 replication in target cells are due to producer cell-dependent alterations to the proteome of HSV-1 virions. LC-MS/MS analysis revealed that virions from different producer cells contain unique and differing quantities of both viral and cellular proteins. In addition, HSV-1 McKrae and HSV-1 strain 17 derived from HaCaTs produce unique virion-associated proteomes relative to one another. From the data provided by the presented experiments, we can conclude that the producer cell has a direct impact on both the complement of proteins associated with HSV-1 virions and the capacity of virions to replicate. Connecting the changes in the proteome to the producer cell-dependent effects on viral replication will require greater analysis of the individual factors that differ between the samples tested.

Critical to assessing the effects of a producer cell on HSV-1 is the evaluation of viral protein incorporation. Several viral proteins were differentially detected in both a producer cell type and strain-dependent manner. In all, 58 viral proteins were identified in purified virions of HSV-1, although one of these proteins, ICP8, was unique to Vero-HSV samples. Viral proteins that were detected at greater quantities (greater than 1 log_2_ fold-change with a *P* < 0.01) in HaCaT-HSV included gG, gK, ICP10, and ICP0. The increased incorporation of ICP0 could explain some of the observed differences in viral replication. ICP0 has multiple activities that include activation of viral gene expression, protein degradation, inhibition of antiviral responses, cell cycle arrest, and inhibition of the DNA damage response (55–61). Knockout or mutations to ICP0 result in significantly less viral replication in cells that have been pre-treated with type I or type II interferon (61). In our experiments, HSV-1 derived from each cell type was sensitive to type I and type II interferon in both HaCaT and HFF-1 cells. However, the greater abundance of ICP0 in HaCaT-HSV may have contributed to the reduced sensitivity of HaCaT-HSV infections to interferon pre-treatment. Interestingly, deletion of ICP0 from HSV-1 results in either severely dampened or a lack of replication in the cornea and trigeminal ganglia of mice. However, this replication is either partially or fully restored in IFN-α/βR^-/-^ or IFN-α/β/γR^-/-^ mice (60). Although differential incorporation of viral proteins into virions is not similar to the complete knockout of a viral gene, it does demonstrate the importance of these proteins *in vivo*, and it is possible that producer cell-dependent changes to protein incorporation impact viral replication and spread *in vivo*. Further testing of the changes to viral protein incorporation will be required to understand if the altered capacity of HSV-1 infection that we have observed is related to the differential incorporation of any of these identified viral proteins.

An alternative interpretation of the cell type differences is that replication is hindered by inclusion of certain viral proteins by Vero-HSV and HFF-HSV. Viral proteins that were incorporated into both Vero-HSV and HFF-HSV at greater than 1 log_2_ fold-change with a *P* < 0.01 in comparison to HaCaT-HSV include pUL24 and pUL42. pUL24 and pUL42 each serve many pro-viral roles within infected cells, including nuclear remodeling, DNA replication, and the inhibition of antiviral responses (62–67). Interestingly, both proteins can bind the NF-κB p50 and p65 subunits and prevent the nuclear translocation of NF-κB (68–71). Although several antiviral roles have been attributed to NF-κB activation, HSV-1 subverts NF-κB function and instead uses it as a pro-viral factor in several cell types, including keratinocytes (72–78). Regions of the HSV-1 genes that encode ICP0 and VP16 each contain κB binding sites, and NF-κB activation is needed for optimal viral gene expression early in infection (75, 77, 79). It is possible that the reduced capacity for productive replication by Vero-HSV and HFF-HSV relative to HaCaT-HSV is due to enhanced cytoplasmic sequestration of NF-κB by the pUL24 and pUL42 proteins that are incorporated into virions at greater abundance.

Evaluation of our LC-MS/MS results needs consideration of prior studies that have evaluated HSV-1 virion-associated proteomes and the factors that complicate direct comparison. The differential incorporation of proteins by HSV-1 virions based on both the strain and producer cell type may explain why the proteins identified in purified HSV-1 virions often vary between studies (50–52) (Table 2). As previously mentioned, Yang and Courtney found that HSV-1 strain KOS purified from Vero and HEp-2 cells contained ICP0 and ICP4, but not virions derived from BHK21 cells (11). However, subsequent studies that purified HSV-1 strain 17 or HSV-1 strain F from BHK21 cells were able to detect ICP0 and ICP4 associated with virions (51, 52, 80). These results allude to the possibility that producer cell-dependent effects on HSV-1 virions can change in a strain-dependent manner. Our analysis detected ICP0 and ICP4 in all treatments, although both proteins were incorporated into virions at different quantities in a producer cell-dependent manner. However, several studies have relied on different methodologies for virion purification and mass spectrometry analysis, which may result in changes to protein detection. In addition, mass spectrometers have become far more sensitive and therefore are more likely to detect peptides that may have been undetected in previous studies (81). Furthermore, it is possible that several of the proteins that have been identified within our LC-MS/MS analysis, as well as other analyses, are not actually associated with HSV-1 virions and could be the result of contaminating proteins or structures. Although our purification scheme utilizes several steps to isolate virions away from extra-virion material, our proteinase k-treated sample revealed that several proteins identified by LC-MS/MS were susceptible to digestion. While a subset of these proteins is likely associated with the virion membrane, it is unlikely that this is the case for all proteins that were digested. In addition, we detected several markers that are known to be associated with extracellular microvesicles such as exosomes.

Interpretation of the producer cell-dependent phenotypes must address changes in the complement of cellular proteins that are detected. Due to the large quantity of cellular proteins that were detected in purified virions, it is unclear as to whether these proteins are specifically incorporated into virions or other structures. Stegen et al. previously used siRNA knockdown experiments to determine that a subset of cellular proteins that are incorporated into HSV-1 are important for replication (82). However, several of these proteins were either not detected in our LC-MS/MS analysis or were incorporated at similar quantities between cell types. Several classes of proteins that were identified have been detected in several viruses, such as translation initiation factors, RNA-binding proteins, and cytoskeletal proteins (8, 50–52, 82–92). Proteins that are known to be associated with exosomes, such as tetraspanins, heat shock proteins, and ESCRT proteins, are commonly identified in purified virions by LC-MS/MS analysis of several viruses, including HSV-1 (8, 50–52, 83, 86, 89, 91, 93). It is possible that extracellular vesicles commonly co-purify with HSV-1 virions, perhaps through aggregation, making it difficult to determine which proteins are truly virion-associated. Previous studies that explicitly isolate exosomes using density ultracentrifugation have not detected HSV-1 DNA within these bands (94, 95). Despite the potential for contamination of our purified virions with extracellular vesicles, it is interesting that the contents of these vesicles may differ based on the producer cell type that is infected. If this is the case, extracellular vesicles excreted during HSV-1 infection may be contributing to the observed differences in HSV-1 replication in infected target cells. Alternatively, it is possible that these proteins that are commonly identified in both exosome and virion purifications are incorporated into HSV-1 virions. A previous study that relied on electron microscopy identified several exosome-associated proteins within mature HSV-1 virions (96). These proteins were specific to exosomes secreted by neurons, none of which have been identified in any previous LC-MS/MS analysis of HSV-1 virions that are isolated from non-neuronal cells. An additional study using a human oligodendroglia cell line identified a fraction of infectious HSV-1 that was secreted in microvesicles (97). Other studies have determined that HSV-1 directly interacts with exocytosis machinery in non-neuronal cells, providing further evidence that HSV-1 utilizes the exocytosis pathway for egress and, as a result, may be incorporating exosome proteins into virions (98). Indeed, further experiments are needed to validate incorporation of these proteins into virions or to determine whether virions are associated with extracellular vesicles.

An alternative, or perhaps complementary hypothesis as to why the producer cell type of HSV-1 alters the replication capacity of virions is that non-infectious particles produced from each cell line are altering replication. These non-infectious particles can include light particles (L-particles) and defective virions. L-particles produced by HSV-1 infected cells contain viral tegument proteins and an envelope but lack a viral capsid and genome (99). Previous work has determined that L-particles can function to antagonize immune responses and enhance viral replication of naked HSV-1 DNA (51, 80, 100). Inquiries into the nature of L-particles have found that the producer cell type can alter the proteomic contents of L-particles, as well as the ratio of L-particles to mature virions that are produced (11, 51). The role of defective virions in HSV-1 infection requires further research, and a wide array of phenotypes has been observed when studying non-infectious virions in the context of infection for several viruses (16–19). These roles can include activating or antagonizing antiviral immunity, enhancing or inhibiting viral replication, and inducing cell death depending on the virus or even viral strain that was observed. Our analysis demonstrated that the HSV-1 genome:PFU ratio was lowest in HaCaT-derived viral inoculum and that HFF-derived inoculum had the highest genome:PFU ratio. By contrast, HFF cells were capable of producing the highest PFU per cell across all tested inocula. Further testing is needed to determine the role of non-infectious virions in the context of HSV-1 infection. However, inhibitory effects by non-infectious virions could contribute to the differences in HSV-1 replication when the inoculum is derived from different producer cells.

A critical question for these and other studies of HSV-1 virions is the relevance to disease. Studies that have looked at virion-associated proteomes of HSV-1 have relied on many cell types, including Vero, BHK-21, and HeLa among others, to propagate material for experiments. The use of different producer cell lines for the propagation of viruses can influence experimental outcomes *in vitro* and *in vivo*. For example, the propagation of SARS-CoV-2 and Ross River virus from Vero cells results in different outcomes of infection in animal models relative to virus that was propagated from more physiologically relevant cell lines (7, 101). These differential outcomes of infection include altered immune responses, viral dissemination, and a reduction in replication. Future experiments are needed to determine whether the producer cell type of HSV-1 influences infection in animal models. In addition, HSV-1 can infect many cell types in humans. The spread of HSV-1 primarily begins with replication in keratinocytes associated with the skin or oral mucosa prior to establishing latency in neurons of the peripheral nervous system. In healthy individuals, HSV-1 spread is generally restricted and is either asymptomatic or manifests as cold sores. However, HSV-1 exhibits a broad tropism in humans, resulting in viral replication within tissues of the eye, liver, and central nervous system, among others, and replication can even result in systemic spread in some patients (14, 102–104). Understanding producer cell-dependent effects on HSV-1 may help us to understand viral spread within and between individuals as well as disease outcomes in patients with severe disease.

## MATERIALS AND METHODS

### Cell culture and viruses

Vero cells purchased from ATCC (CCL-81), HaCaT cells purchased from AddexBio Technologies (NC0309203), and HFF-1 cells purchased from ATCC (SCRC-1041) were grown in Dulbecco’s modified Eagle’s medium (DMEM) supplemented with 10% fetal bovine serum (FBS) and 1% penicillin/streptomycin (pen/strep) at 37°C in 5% CO_2_. HSV-1 strain McKrae was provided by Dr. William Halford. HSV-1 strain 17 was provided by Dr. Lynn Enquist. Prior to propagation for experiments, both HSV-1 strains were isolated by plaque purification and amplified on HaCaT cells to produce a genetically homogenous high-titer master stock. The master stock was then inoculated onto three biological replicate HaCaT, Vero, and HFF-1 cell cultures at an MOI of 0.01. HSV-1 was harvested from each cell line upon observation of full cytopathic effect at 48 h (HaCaT), or 72 h (Vero and HFF-1) for use in downstream experiments to compare producer cell effects. HSV-1 was grown in DMEM supplemented with 2% FBS and 1% pen/strep. All HSV-1 stocks were titered by plaque assay on Vero cells.

### Fluorescence microscopy

Epifluorescence imaging was performed on a Nikon Ti-Eclipse inverted microscope (Nikon Instruments) equipped with a Spectra X LED excitation module (Lumencor) and fast-switching emission filter wheels (Prior Scientific). Fluorescence imaging used paired excitation/emission filters and dichroic mirrors for DAPI, green fluorescent protein (GFP), and tetramethylrhodamine (TRITC) filters (Chroma Corp.). All brightfield images were acquired using phase-contrast configuration at 4× or 20× optical magnification. Imaging immunofluorescent-stained samples were set to the same exposure and power when comparing treatments within the same sampling group as determined by cell type and staining time post-infection.

### Quantification of HSV-1 antigen

Cells were infected with HSV-1 McKrae using an MOI of 1 or 10. Cells were washed with DPBS 1 hpi and replaced with DMEM supplemented with 2% FBS and 1% pen/strep. Cells were fixed at 3 or 6 hpi as described above. Immunofluorescent stains of HSV-1 antigen in infected cells were quantified for fluorescent intensity using Fiji ImageJ. One image from each of three biological replicates was selected for analysis at each time point. All of the cells within the image were selected as an independent region of interest (ROI). The mean gray value was measured for each ROI and background noise subtracted based on the mean gray value of mock-infected cells.

### Purification of HSV-1 virions

HSV-1 McKrae was purified from HaCaT, Vero, and HFF-1 cells using a modified purification scheme that was previously described (52). Cells were infected with HSV-1 McKrae at an MOI of 0.01. Inoculum was removed 1 hpi. Cells were washed with DPBS and replaced with serum-free DMEM. HSV-1 was propagated and harvested upon observation of full cytopathic effect at 48 h (HaCaT) or 72 h (Vero and HFF-1). Viral supernatants were harvested and centrifuged at 300 × *g* for 10 min. The supernatant was then filtered through a 0.45 µm filter. Virions were pelleted by centrifugation at 20,000 × *g* for 30 min at 4°C. Virions were resuspended in PBS, sonicated, and incubated with 100 U/mL of DNAse I at 37°C for 1 h. Virions that received proteinase k treatment were incubated with 0.1 mg/mL of Proteinase K for 45 min at room temperature and subsequently treated with 2 mM of phenylmethylsulfonyl fluoride (PMSF). Samples were further diluted in DPBS, loaded onto a 10% ficoll 400 cushion, and centrifuged at 26,000 RPM for 2 h at 4°C on an SW41 Ti rotor. The pellets were resuspended in 100 μL of 0.75% NP-40 for 1 h on ice. Purified HSV-1 was stored at −80°C. This same protocol was used for HSV-1 strain 17 purified from HaCaT cells and mock-infected HaCaT, Vero, and HFF-1 cells that were scraped and frozen at −80°C prior to purification.

### Quantification of viral transcripts by qRT-PCR

Cells that were infected with HSV-1 at an MOI of 10 were harvested in 0.4 mL of Trizol reagent and immediately stored at −20°C. RNA was isolated and purified according to the Trizol manufacturer’s protocol and resuspended in molecular biology-grade water. Isolated RNA was treated with DNAase to assure that samples were free of DNA contamination using the DNA-free Kit (Invitrogen AM1906) in accordance with the manufacturer’s protocol. RNA concentration and quality were assessed by 260/280 nm absorbance using an ND1000 UV/Vis NanoDrop (S/N 6612). 1 µg of RNA was reverse-transcribed using M-MuLV Reverse Transcriptase (NEB MO253L) in accordance with the manufacturer’s protocol. No RT control reactions were performed to ensure the absence of contaminating DNA in isolated RNA preparations. cDNA was stored at −20°C. qPCR was performed using the Power Sybr Green kit following the manufacturer’s protocol (applied biosystems 4367659). Primers targeted viral ICP27, ICP8, and VP5, and cellular 28S rRNA was targeted as an endogenous control.

### HSV-1 genome quantification using qPCR

Isolation of HSV-1 DNA for qPCR standard curve: HSV-1 McKrae genome was concentrated and isolated to form a standard curve for qPCR quantification of HSV-1 viral genomes from viral stocks. To do this, HSV-1 supernatants were harvested from HaCaT cells infected with HSV-1 McKrae at an MOI of 10 for 20 h. Supernatants were centrifuged at 300 × *g* for 10 min and filtered through a 0.45 µm filter. Virions were then pelleted by centrifugation at 20,000 × *g* for 30 min at 4°C. Virions were resuspended in PBS, sonicated, and incubated with 100 U/mL of DNAse I at 37°C for 1 h to remove contaminating cellular DNA. HSV-1 genome was then isolated from digested virions using the Zymo Quick-DNA miniprep kit (cat# D3024). The HSV-1 viral genome concentration was calculated using nanodrop prior to building the standard curve for qPCR analysis.

Measurement of HSV-1 genome:PFU content: HSV-1 virion-associated genome quantitation was calculated by comparing HSV-1 genome content to known standard curve values of HSV-1 genomic DNA. Prior to qPCR analysis, the three biological replicate stocks derived from HaCaT, Vero, and HFF-1 cells that were used in experiments were treated with DNAse I and processed through the Zymo Quick-DNA miniprep kit in accordance with the manufacturer’s protocol. qPCR analysis was performed using the Power SYBR Green kit (Applied Biosystems, cat no. 4367659), targeting HSV-1 ICP27. 28S rRNA was targeted as quality control to ensure no cellular DNA was present in standard curve or sample DNA.

qPCR primers were as follows:

28S fwd 5′-GGG CCG AAA CGA TCT CAA CC-3′

28S rev 5′-GCC GGG CTT CTT ACC CAT T-3′

ICP27 fwd 5′-AGA CGC CTC GTC CGA CGG-3′

ICP27 rev 5′-AGG CGC GAC CAC ACA CTG T-3′

ICP8 fwd 5′-CAT CAG CTG CTC CAC CTC GCG-3′

ICP8 rev 5′-GCA GTA CGT GGA CCA GGC GGT′3′

VP5 fwd 5′-TGG ATG GTA TGG TCC AGA TGC-3′

VP5 rev 5′-GCA CAA CGG CGC TGC TCT-3′

### Fixation and immunofluorescent staining

Cells were washed with DPBS and fixed with ice-cold 50:50 methanol:acetone for 20 minutes at RT. Methanol:acetone was removed, dried, and cells were blocked with 2% bovine serum albumin (BSA) in DPBS for 10 min at RT. Cells were incubated with rabbit polyclonal HSV-1 anti-serum (1:500, Dako) diluted in antibody solution (1% BSA in DPBS) for 1 h at RT. Cells were washed 3× with DPBS and incubated with Donkey anti-Rabbit IgG Alexa Fluor 488 (2 drops/mL) diluted in antibody solution for 1 h at RT. Cells were washed with DPBS and incubated with 1:5,000 Hoechst in DPBS for 10 min. Cells were washed 2× with DPBS prior to fluorescent imaging.

### Growth curves

Cells were infected with HSV-1 McKrae at an MOI of 10 or 1. The inoculum was removed 1 hpi, and cells were washed with DPBS and incubated in cell media consisting of DMEM supplemented with 2% FBS and 1% pen/strep at 37°C in 5% CO_2_. Cells and supernatants were harvested by scraping at corresponding time points. Samples were stored at −80°C and sonicated after thawing for measurement of viral titers by plaque assay on Vero cells. For experiments in which interferon pre-treatment was used, cells were treated with 100 U/mL (HaCaT) or 1,000 U/mL (HFF-1) of IFN-β or IFN-γ. Interferon was removed from cells after 16 hours of incubation, washed with DPBS, and infected as described above.

### Liquid chromatography-mass spectrometry

Total protein from each sample was reduced, alkylated, and digested using single-pot, solid-phase-enhanced sample preparation with sequencing grade modified porcine trypsin (Promega) (105). Tryptic peptides were trapped and eluted on 3.5 μm CSH C18 resin (Waters) (4 mm × 75 μm) then separated by reverse phase XSelect CSH C18 2.5 μm resin (Waters) on an in-line 150 × 0.075 mm column using an UltiMate 3000 RSLCnano system (Thermo). Peptides were eluted at a flow rate of 0.300 μL/min using a 60 min gradient from 98% Buffer A (0.1% formic acid, 0.5% acetonitrile):2% Buffer B (0.1% formic acid, 99.9% acetonitrile) to 95:5 at 2.0 minutes to 80:20 at 39.0 minutes to 60:40 at 48.0 minutes to 10:90 at 49.0 minutes and hold until 53.0 minutes and then equilibrated back to 98:2 at 53.1 minutes until 60 minutes. Eluted peptides were ionized by electrospray (2.4 kV) through a heated capillary (275°C) followed by data collection on an Orbitrap Exploris 480 mass spectrometer (Thermo Scientific).

Precursor spectra were acquired with a scan from 385 to 1,015 Th at a resolution set to 60,000 with 100% AGC, max time of 50 msec, and an RF parameter at 40%. DIA was configured on the Orbitrap 480 to acquire 50 × 12 Th isolation windows at 15,000 resolution, normalized AGC target 500%, maximum injection time 40 ms) (106). A second DIA was acquired in a staggered window (12 Th) pattern with optimized window placements. Following data acquisition, data were searched using Spectronaut (Biognosys version 19.1) against the UniProt database for *Homo sapiens* (proteome ID UP000005640), *Chlorocebus sabaeus* (proteome ID UP000029965)*,* HHV1 strain 17 (proteome ID UP000009294), and the NCBI database for HHV1 McKrae (GenBank: JX142173.1) (April 2024), respectively, using the directDIA method with an identification precursor and protein q-value cutoff of 1%, generate decoys set to true, the protein inference workflow set to maxLFQ, inference algorithm set to IDPicker, quantity level set to MS2, cross-run normalization set to false, and the protein grouping quantification set to median peptide and precursor quantity. Protein MS2 intensity values were assessed for quality using ProteiNorm (107). The data were normalized using Variance-stabilizing normalization (VSN) to bring the samples to the same scale by first performing a transformation to remove variance caused by systematic experimental factors and then applying a generalized log2 transformation (108–110). Data were analyzed using proteoDA to perform statistical analysis using Linear Models for Microarray Data (limma) with empirical Bayes (eBayes) smoothing to the standard errors (108, 111–115). Proteins with an FDR-adjusted *P*-value < 0.05 and a fold-change >2 were considered significant.

## Data Availability

All data supporting the work presented here are available upon request. The protein identification scores table from the LC-MS/MS analysis of purified virions has been supplied as supplemental material (Table S1). In addition, the full range of normalized protein scores has been supplied as supplemental material (Tables S2 and S3). Any additional information and clarification can be requested from the corresponding author.

## SUPPLEMENTAL MATERIAL

Table S1 jvi.00829-25-s0001.csv Viral and cellular proteins detected by LC-MS/MS in all treatment groups.

Table S2 jvi.00829-25-s0002.xlsx Normalized protein scores and pairwise comparisons of relative detection from purified HSV-1 virions.

Table S3 jvi.00829-25-s0003.xlsx Proteins identified in control samples and comparisons between cell types and HSV-1 strains.

ASM does not own the copyrights to Supplemental Material that may be linked to, or accessed through, an article. The authors have granted ASM a non-exclusive, world-wide license to publish the Supplemental Material files. Please contact the corresponding author directly for reuse.
